# FgGCV1, a glycine cleavage system T protein, regulates glycine metabolism and sexual reproduction in *Fusarium graminearum*

**DOI:** 10.3389/fpls.2026.1771151

**Published:** 2026-03-18

**Authors:** Jie Wang, Ling Yang, Enhui Bai, Banpu Ruan, Fei Chen, Yanli Zhang, Limin Wu, Yanchun Yu

**Affiliations:** 1 College of Life and Environmental Sciences, Hangzhou Normal University, Hangzhou, China; 2 College of Life Sciences, South China Agricultural University, Guangzhou, China

**Keywords:** FgGCV1, *Fusarium graminearum*, glycine cleavage system, glycine metabolism, sexual reproduction

## Abstract

Fusarium head blight (FHB), caused by several *Fusarium* species, among which the most important and widely distributed worldwide is *Fusarium graminearum*, in the case that the causal agent is *F. graminearum*, FHB spread is closely linked to the pathogen’s sexual reproduction. The T protein of the glycine cleavage system (GCVT) is a key component of carbon and nitrogen metabolism in organisms, however its biological function in filamentous fungi, particularly *F. graminearum*, is still unclear. In this study, we characterized two GCVT homologs (*FgGCV1* and *FgGCV2*) to obtain a better understanding of the metabolic processes occurring in *F. graminearum*. We found that FgGCV1 and FgGCV2 are localized in mitochondria. Deletion of *FgGCV2* had no obvious phenotypic alterations, whereas Δ*FgGCV1* mutant exhibited severe defects in sexual reproduction. Notably, the sexual reproduction defect in the Δ*FgGCV1* mutant was completely restored by exogenous addition of 5,10-methylenetetrahydrofolate (5,10-CH_2_-THF). Moreover, Δ*FgGCV1* accumulated higher intracellular glycine contents and exhibited increased tolerance to calcium stress. Transcriptome analysis identified 1,482 differentially expressed genes (DEGs) in the Δ*FgGCV1* mutant, with DEGs enriched in glycine, serine, and threonine metabolism, as well as reproductive and developmental processes. Collectively, our findings demonstrate that *FgGCV1* plays a crucial role in regulating glycine metabolism and sexual reproduction in *F. graminearum* through the glycine cleavage system (GCS) pathway, providing new insights into the molecular mechanisms underlying the pathogen’s metabolic regulation and sexual development.

## 1 Introduction

Fusarium head blight (FHB) is a complex fungal disease caused by multiple *Fusarium* species, with *F. graminearum* being the most geographically widespread and virulently aggressive species worldwide (Jian et al., 2022; Sun et al., 2023). This pathogen not only causes significant yield losses, but also contaminates grains with harmful mycotoxins, such as deoxynivalenol (DON) and zearalenone, posing serious threats to food and feed safety (Chen et al., 2019). The epidemiology of FHB caused by *F. graminearum* is intrinsically linked to the sexual reproduction of this pathogen (Trail et al., 2002; Trail, 2007), while the sexual reproductive mechanisms of other FHB-causing *Fusarium* species remain uncharacterized in nature. The epidemiology of FHB is intrinsically linked to the sexual reproduction of *F. graminearum* (Trail et al., 2002; Trail, 2007). During host flowering, mature fruiting bodies (perithecia) release ascospores, which are dispersed by wind or rain splash to serve as the primary inoculum for new infections (Luo et al., 2014; Ding et al., 2023). Thus, elucidating the genetic regulators of sexual reproduction in *F. graminearum* could reveal novel targets for integrated FHB management strategies.

The glycine cleavage system (GCS), a conserved multienzyme complex present in organisms ranging from bacteria to humans, is central to one-carbon metabolism. GCS catalyzes the reversible oxidation of glycine, producing carbon dioxide, ammonia, 5,10-methylenetetrahydrofolate (5,10-CH2-THF), and reduced pyridine nucleotides (Fox and Stover, 2008; Kikuchi et al., 2008; Radha Rama Devi et al., 2018). Glycine is a non-essential amino acid that is synthesized from serine and degraded via GCS (Kikuchi et al., 2008). This mitochondrial system comprises four core components: P-protein (glycine decarboxylase), H-protein (hydrogen carrier protein), T-protein (aminomethyltransferase) and L-protein (dihydrolipoamide dehydrogenase) (Radha Rama Devi et al., 2018). The T-protein is particularly critical, as it catalyzes the release of ammonia from the aminomethyl moiety bound to the lipoate cofactor of the H-protein of the complex and the transfer of a one-carbon unit to tetrahydrofolate, generating 5,10-CH2-THF (Kikuchi, 1973; Kikuchi et al., 2008; Radha et al., 2018). Dysfunction of T protein disrupts the entire GCS system, leading to severe physiological consequences across kingdoms. In humans, mutations in the T-protein-encoding *AMT* gene cause non-ketotic hyperglycinemia (NKH), a lethal neurological disorder characterized by glycine accumulation. This accumulation leads to severe neonatal encephalopathy, intractable epilepsy, profound mental retardation, and life-threatening respiratory depression (Hayasaka et al., 1993; Zhou et al., 2022). In plants, *Arabidopsis thaliana* T-protein mutants (*gcvT*) exhibit severe photorespiratory defects and are non-viable under ambient air conditions (Timm et al., 2018). Similarly, the *Saccharomyces cerevisiae* T-protein mutant (*GCV1*) cannot utilize glycine as a nitrogen source and lacks GCS activity (McNeil et al., 1997). In the bacterium *Streptomyces griseus* knockout of *gcvT* also leads to a complete loss of glycine cleavage activity, substantial intracellular glycine accumulation, and a severe growth defect (Tezuka et al., 2014). Despite its established importance in other systems, the function of GCS T-protein in filamentous fungi remains largely unexplored, and its potential role in fungal pathogenesis and development is unknown.

Given the largely uncharacterized biological functions of the glycine cleavage system T-protein (GCVT) in filamentous fungi and its unknown regulatory roles in *F. graminearum* metabolism and developmental processes, the present study aimed to systematically characterize the two GCVT homologs (FgGCV1 and FgGCV2) in *F. graminearum*. We first conducted bioinformatic and molecular analyses to clarify their sequence features, subcellular localization and spatiotemporal expression patterns. Then, gene deletion and complementation strains were constructed to investigate their biological functions in fungal growth, stress response and sexual reproduction. Further, we combined metabolite detection, exogenous substance complementation and transcriptome sequencing to elucidate the molecular mechanism underlying *FgGCV1*-mediated regulation of glycine metabolism and sexual reproduction. This study is expected to fill the knowledge gap of GCVT function in *F. graminearum* and reveal the link between fungal one-carbon metabolism and sexual development, providing novel insights into the metabolic regulation network of *F. graminearum*.

## 2 Materials and methods

### 2.1 Fungal strains and culture conditions

*Fusarium graminearum* strain PH-1 (NRRL 31084) was used as the parental wild-type (WT) in this study. For colony morphology and colony diameter measurement, all strains were cultured at 25°C on potato dextrose agar (PDA), minimal medium (MM), and complete medium (CM) for 3 days. Colony diameters were measured for each strain after culturing for 3 days at 25°C. To induce asexual reproduction, conidia were incubated in liquid carboxymethylcellulose (CMC) medium (1 g NH_4_NO_3_, 1 g KH_2_PO_4_, 0.5 g MgSO_4_·7H_2_O, 1 g yeast extract, 15 g CMC, and 1 L distilled water) with shaking at 28°C for 5 days in a rotary shaker (200 rpm). Conidial concentrations were determined using a hemocytometer. Fungal mycelia were harvested from yeast extract peptone dextrose (YEPD) (0.3% yeast extract, 1% peptone, 2% dextrose) liquid medium for total genomic DNA and RNA extraction. To assess the sensitivity of the Δ*FgGCV1* mutant to various stresses, mycelial growth was evaluated on PDA plates supplemented with (or without) 1 M NaCl, 0.05% SDS, 0.2 M CaCl_2_, 0.3 g/L Congo red, or 0.02% H_2_O_2_. For sexual reproduction induction, aerial hyphae of 7-day-old carrot agar cultures were pressed down with a sterile glass rod, followed by the addition of 1 mL sterile 2.5% Tween 20 solution per plate. Perithecium formation, cirrhi production, asci development, and ascospore discharge were examined as previously described (Wang et al., 2022b; Zeng et al., 2018).

### 2.2 Construction of two FgGCV deletion mutants and FgGCV1 complementation strains

The split-marker method (Catlett et al., 2003) was used to construct *FgGCV1* and *FgGCV2* deletion mutants. Taking *FgGCV1* as an example, the 0.8 kb upstream and 0.8 kb downstream flanking sequences of *FgGCV1* were amplified by PCR from *F. graminearum* PH-1 genomic DNA. The resulting PCR products were fused with the hygromycin phosphotransferase (hph) resistance gene cassette via overlapping PCR and transformed into PH-1 protoplasts as described previously (Wang et al., 2022b). The same method was used to generate *FgGCV2* deletion mutants. Transformants were selected on PDA plates containing 225 μg/mL hygromycin B. For complementation assays, the full-length *FgGCV1* gene, including its native promoter region, was amplified by PCR using primer pairs *FgGCV1*-CF/CR and cloned into the vector pKNTG to generate the complementary construct (Wang et al., 2022a). The recombinant plasmid was transformed into Δ*FgGCV1* protoplasts to generate Δ*FgGCV1*-C complemented strains, which were selected on medium supplemented with 200 μg/mL G418 and 225 μg/mL hygromycin B. The *FgGCV1* and *FgGCV2* deletion mutants were identified by PCR and RT-PCR. All primers used in this study are listed in **Supplementary Table 3**.

### 2.3 Pathogenicity assay

Pathogenicity assays on wheat heads were conducted as described previously (Wang et al., 2022b). Briefly, a 10 μL aliquot of conidial suspension (1×105 conidia/mL) was injected into a floret in the middle spikelet of flowering wheat heads of the susceptible cultivar Jimai 22. The inoculated wheat heads were incubated at 25°C under 95%-100% relative humidity. The experiment was performed with eight replicate per strain. Fifteen days post-inoculation, the number of infected spikelets per inoculated wheat head was recorded and photographed.

### 2.4 Staining and microscopic observation

For subcellular localization analysis, MitoTracker Red CMXRos (Invitrogen) was added to mycelial cultures to a final concentration of 1 μM. After incubation in the dark for 20 min, mycelia were washed twice with phosphate-buffered saline (PBS). Microscopic observations were performed using a laser scanning confocal microscope (LSM 880 NLO, Zeiss, Germany).

### 2.5 RNA extraction and quantitative reverse transcription PCR

For asexual stage samples, RNA was isolated from 24 h YEPD cultures (mycelia) and 24 h CMC cultures (sporulation). For sexual development samples, RNA was extracted from 7-day-old carrot agar hyphae (0 day post self-crossing, 0 dps) and 3-, 5-, and 7-day-old perithecia (3, 5, and 7 dps). Total RNA was extracted using TRIzol reagent (Cowin Biotech, Taizhou, China) according to the manufacturer’s instructions. First-strand cDNA was synthesized using a cDNA synthesis kit (Cowin Biotech, Taizhou, China), and RT-qPCR was performed with SuperStar Universal SYBR Master Mix (Cowin Biotech) on a CFX96 Real-Time PCR Detection System (Bio-Rad, Hercules, CA, USA). Relative transcript levels were quantified using the comparative 2^^-^ΔΔCt^ method, with the *Actin* gene serving as the internal reference (Livak and Schmittgen, 2001). Primers used for RT-qPCR are listed in **Supplementary Table 3**.

### 2.6 RNA-seq analysis

Seven-day-old perithecia of PH-1 and Δ*FgGCV1* mutant were harvested from carrot agar cultures, and total RNA was extracted using TRIzol reagent (Cowin Biotech, Taizhou, China). Three independent biological replicates were prepared for each strain. Library construction and sequencing were performed on an Illumina HiSeq 2500 platform by Tsingke Co., Ltd. (Beijing, China), with each library generating at least 24 Mb of paired-end reads. Raw reads were mapped to the *F. graminearum* PH-1 reference genome using HISAT2 (v2.2.0). Read counts for each gene were calculated using DESeq2 (v1.26.0). Genes with a false discovery rate (FDR) < 0.01 and |log_2_fold-change| > 1 were considered differentially expressed genes (DEGs). Transcripts were assembled from RNA-seq mappings of all samples using StringTie (v2.1.2). The RNA-seq data have been deposited in the NCBI Sequence Read Archive (SRA) database under accession number SRR36300736 to SRR36300741.

### 2.7 Intracellular glycine content measurement

Mycelia were collected from 2-day-old YEPD cultures, and 100 mg of mycelial samples were ground to a fine powder in liquid nitrogen. Intracellular glycine content was determined using a commercial Glycine Assay Kit (Keaibo Biotechnology Inc., Shanghai, China). All measurements were performed with three independent biological replicates.

## 3 Results

### 3.1 Identification of glycine cleavage system T genes in *F. graminearum*

**Figure 1:**
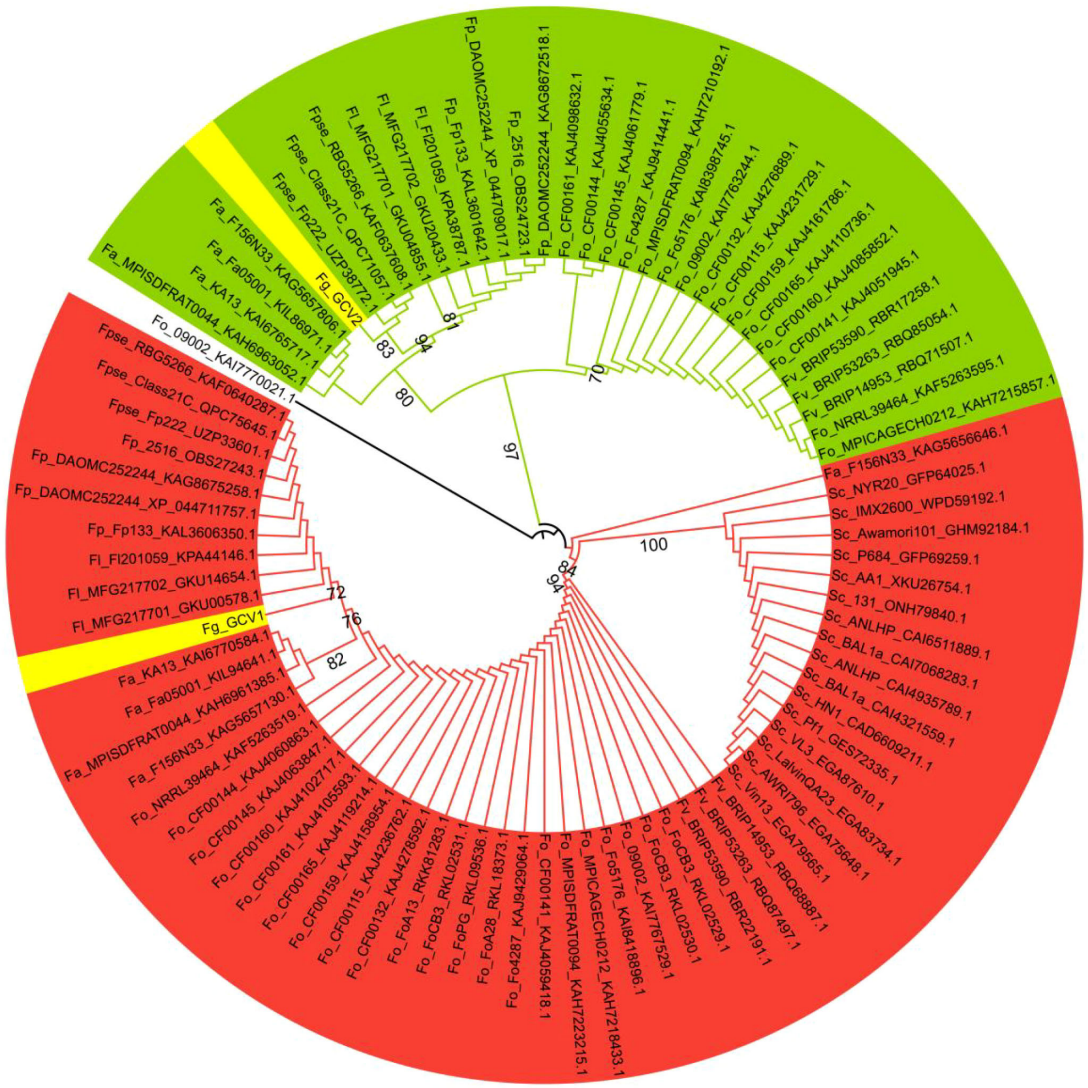
Phylogenetic tree analysis of glycine cleavage system T genes. The circular phylogenetic tree is divided into two major clades (green and red) with distinct taxonomic affiliations. The tree was constructed using the maximum-likelihood (ML) method in RAxML 8.2.10, and amino acid sequences were retrieved from the GenBank database. The branches’ names are presented in the format of species abbreviation_strain identifier_NCBI gene accession number. Fp, *Fusarium poae*; Fa, *Fusarium avenaceum*; Fo, *Fusarium oxysporum*; Fpse, *Fusarium pseudograminearum*; Fg, *Fusarium graminearum*; Fl, *Fusarium langsethiae*; Fv, *Fusarium verticillioides*; Sc, *Saccharomyces cerevisiae*.

**Figure 2:**
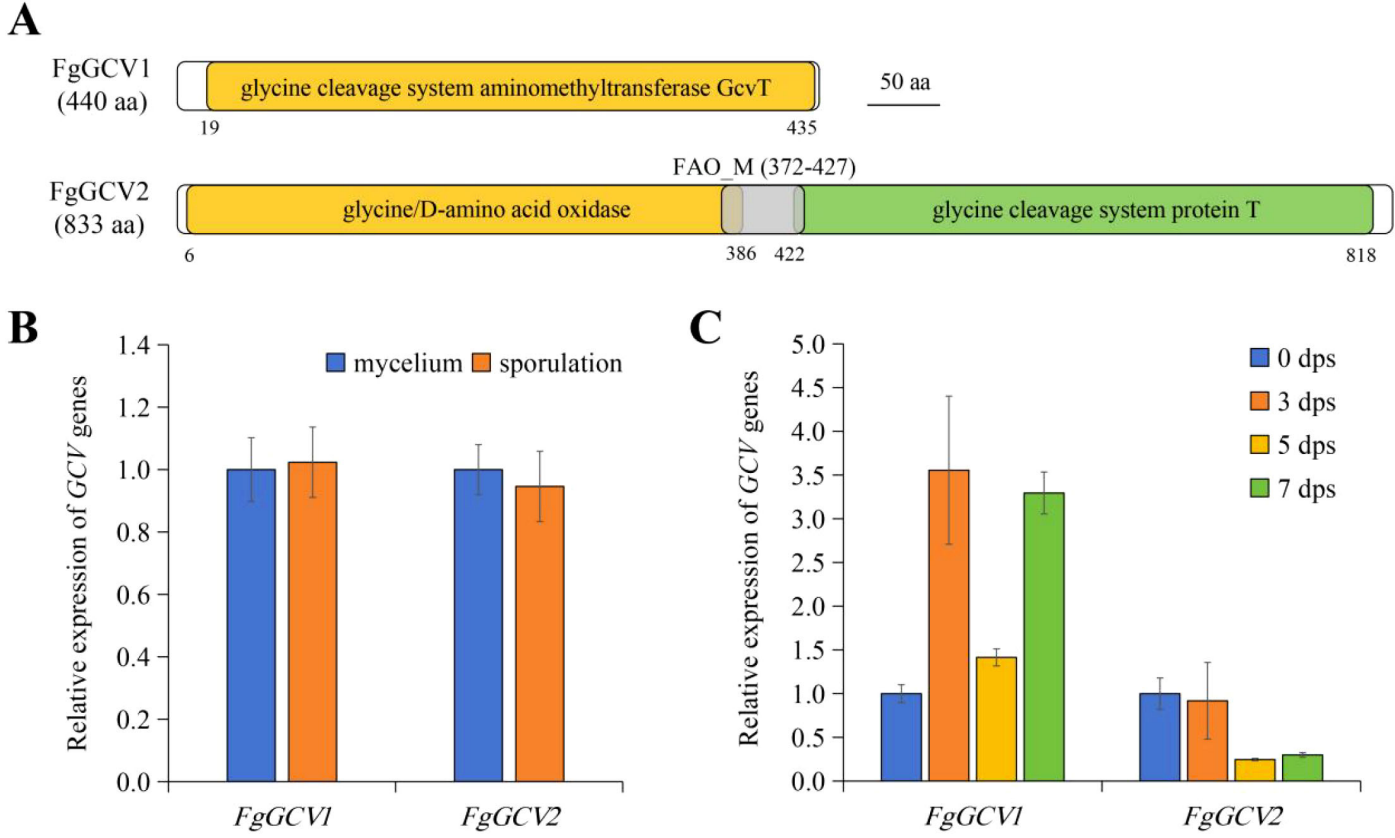
Domain structure and expression patterns of *FgGCV1* and *FgGCV2*. **(A)** Domain structures of *FgGCV1* and *FgGCV2*. **(B)** The expression patterns of *FgGCV1* and *FgGCV2* in asexual stages. RNA was extracted from 24 h YEPD cultures (mycelia) and 24 h CMC cultures (conidiation). **(C)** The expression patterns of *FgGCV1* and *FgGCV2* in sexual stage. RNA was extracted from 7-day-old carrot agar hyphae (0 day post self-crossing, 0 dps) and 3-, 5-, 7-day-old perithecia (3, 5, 7 dps). Relative transcript levels were determined by RT-qPCR using the *Actin* gene as an internal reference, and data are presented as mean ± standard deviation (SD) from three independent biological replicates.

To investigate the glycine cleavage system (GCS) in *F. graminearum*, we identified two genes encoding putative T-proteins (aminomethyltransferases), designated *FgGCV1* (*FGSG_ 01151*) and *FgGCV2* (*FGSG_03414*). A phylogenetic analysis revealed that *FgGCV1* and *FgGCV2* cluster closely with homologs from other *Fusarium* species, including *Fusarium oxysporum*, *Fusarium pseudograminearum*, and *Fusarium avenaceum* (**Figure 1**). Notably, *FgGCV1* groups with homologs from *S. cerevisiae*, whereas *FgGCV2* forms a distinct, highly conserved clade with *F. oxysporum*, suggesting potential functional divergence. Domain architecture analysis highlighted fundamental structural differences between the two proteins (**Figure 2A**). FgGCV1 (440 aa) contains a single GCVT domain, consistent with canonical T-proteins. In contrast, FgGCV2 (833 aa) is a larger, multi-domain protein, featuring an N-terminal glycine/D-amino acid oxidase domain, a central FAO_M domain, and a C-terminal GCVT domain. This complex structure suggests FgGCV2 may have functions beyond its role in the core GCS complex.

Further analysis of their physicochemical properties revealed distinctions consistent with their divergent structures (**Supplementary Table 1**). FgGCV1 is a 47.11 kDa basic protein (pI 8.52), while FgGCV2 is a 91.41 kDa acidic protein (pI 6.56), indicating they likely operate in different cellular microenvironments. Both proteins are hydrophilic, as evidenced by negative GRAVY scores, which aligns with their predicted mitochondrial localization. Secondary structure prediction also revealed their domain differences, with FgGCV2 exhibiting a higher α-helix content compared to FgGCV1, potentially associated with its additional domains (**Supplementary Figure 1**).

We next examined the expression patterns of *FgGCV1* and *FgGCV2* across different growth and developmental stages using RT-qPCR. Their transcription levels showed no significant variation during mycelium and conidiation (**Figure 2B**). Strikingly, their expression diverged markedly during sexual reproduction. Following self-crossing, *FgGCV1* expression was induced, peaking at 3 days post-selfing (dps). In contrast, *FgGCV2* expression decreased over the same period (**Figure 2C**). These distinct temporal expression profiles strongly suggest that *FgGCV1* and *FgGCV2* have non-redundant functions, with *FgGCV1* being specifically implicated in the sexual development of *F. graminearum*.

### 3.2 Subcellular localization of FgGCV1 and FgGCV2

**Figure 3:**
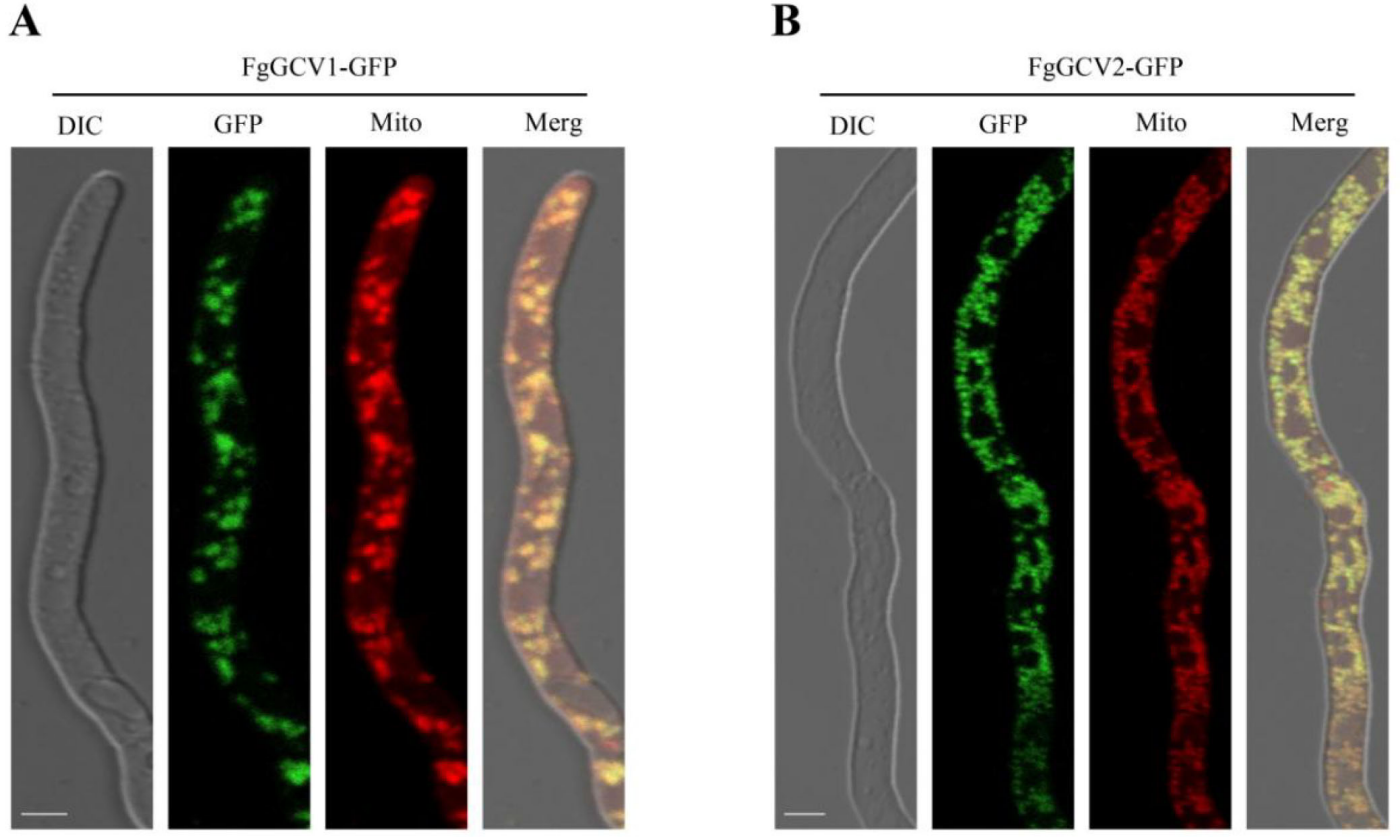
Subcellular localization of *FgGCV1***(A)** and *FgGCV2***(B)** in *F. graminearum.* Mycelia of wild-type PH-1 expressing FgGCV1-GFP and FgGCV2-GFP fusion proteins were stained with MitoTracker (mitochondrial marker) and examined under a laser scanning confocal microscope. Bar=5 μm.

To determine the subcellular localization of FgGCV1 and FgGCV2, we generated strains expressing FgGCV1-GFP and FgGCV2-GFP fusion proteins under the control of their native promoters. The pFgGCV1-GFP and pFgGCV2-GFP transformed strains were stained with the MitoTracker Red CMXRos prior to confocal microscopy, respectively. The results showed that fluorescence signals from FgGCV1-GFP and FgGCV2-GFP overlapped completely with the mitochondrial marker (MitoTracker) in mycelia (**Figure 3**). This confirms the mitochondrial localization of both proteins, which is consistent with the established role of the GCS and indicates the conservation of this localization in fungi.

### 3.3 Construction and validation of FgGCV1 and FgGCV2 deletion mutants

To investigate the biological functions of *FgGCV1* and *FgGCV2*, deletion mutants (Δ*FgGCV1*, Δ*FgGCV2*) were constructed via homologous recombination strategy (**Supplementary Figures 2A,C**), and their successful generation was verified by PCR and RT-PCR (**Supplementary Figures 2B, D–F**). A complemented strain (Δ*FgGCV1-C*) was also generated to confirm the phenotypic defects in Δ*FgGCV1* were caused by the deletion of *FgGCV1*. During asexual development, the *FgGCV1* and *FgGCV2* mutants exhibited no discernible defects in hyphal growth, conidiation, or pathogenicity on wheat when compared to the wild-type PH-1 and complemented strain (**Supplementary Figure 3**; **Supplementary Table 2**). These results demonstrate that neither *FgGCV1* nor *FgGCV2* is essential for these core vegetative and pathogenic processes in *F. graminearum*.

### 3.4 FgGCV1 regulates the sexual reproduction of *F. graminearum*

**Figure 4:**
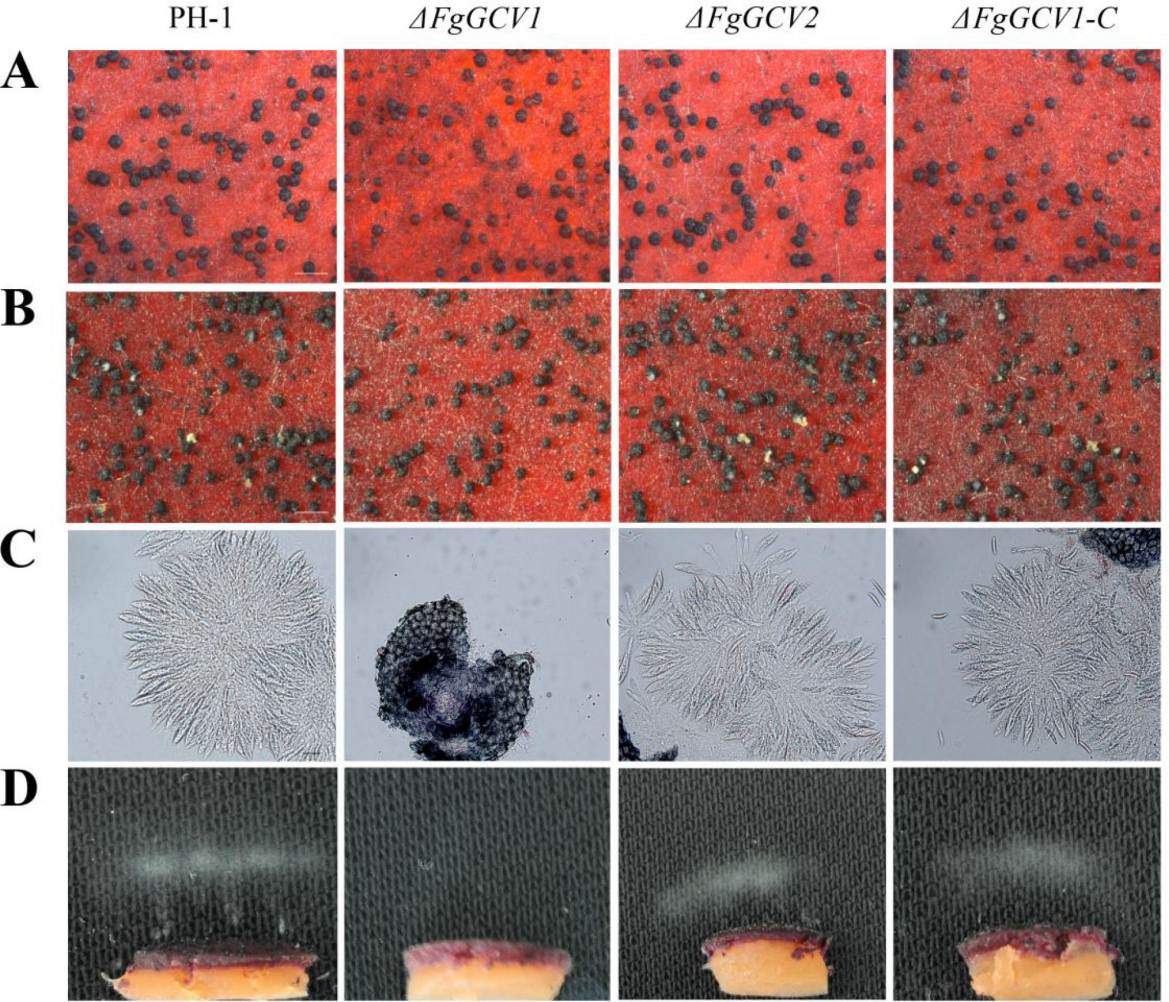
Effects of *FgGCV1* and *FgGCV2* on sexual development. **(A)** Morphology of perithecia of the wild-type PH-1, Δ*FgGCV1*, Δ*FgGCV2* and Δ*FgGCV1-C* strains were observed on carrot agar medium. Photographs were captured at 7 days post-fertilization (dpf). **(B)** Ascospore cirrhi (yellow extrusions from perithecium ostioles) were imaged at 14 dpf. Bar=500 µm. **(C)** Asci morphology from cracked perithecia were examined at 7 dpf. Bar=20 µm. **(D)** Ascospore discharge was examined using 7-day-old perithecia. Photographs were taken after being released for 18 h The white cloud represents an accumulation of discharged ascospores.

Sexual reproduction is a critical step in the infection cycle of *F. graminearum*. To determine whether *FgGCV1* and *FgGCV2* are involved in this process, we assessed the sexual reproduction capabilities of corresponding mutants. On carrot agar medium, the wild-type (PH-1), Δ*FgGCV2*, and Δ*FgGCV1*-*C* strains all formed abundant, normal-shaped perithecia by 7 days post-fertilization (dpf) (**Figure 4A**). In contrast, although the Δ*FgGCV1* mutant also produced perithecia, it exhibited significantly fewer ascospore cirrhi (**Figure 4B**), indicating a defect in ascospore release. To further investigate this phenotype, we examined asci development and ascospore discharge using established methods (Wang et al., 2022b). After 7 days of sexual induction, mature asci were readily observed within perithecia of PH-1, Δ*FgGCV2*, and Δ*FgGCV1-C* strains. However, the Δ*FgGCV1* mutant failed to produce any asci even after 4 weeks of induction (**Figure 4C**). Consistent with this, forcible discharge of ascospores occurred abundantly in the wild-type strain following 18 h of incubation, whereas no ascospore discharge was detected in Δ*FgGCV1* (**Figure 4D**). Taken together, these findings reveal that *FgGCV2* is dispensable for sexual reproduction in *F. graminearum*, while *FgGCV1* is essential for normal ascus development and ascospore formation.

### 3.5 The ΔFgGCV1 mutant exhibits enhanced tolerance to calcium stress

**Figure 5:**
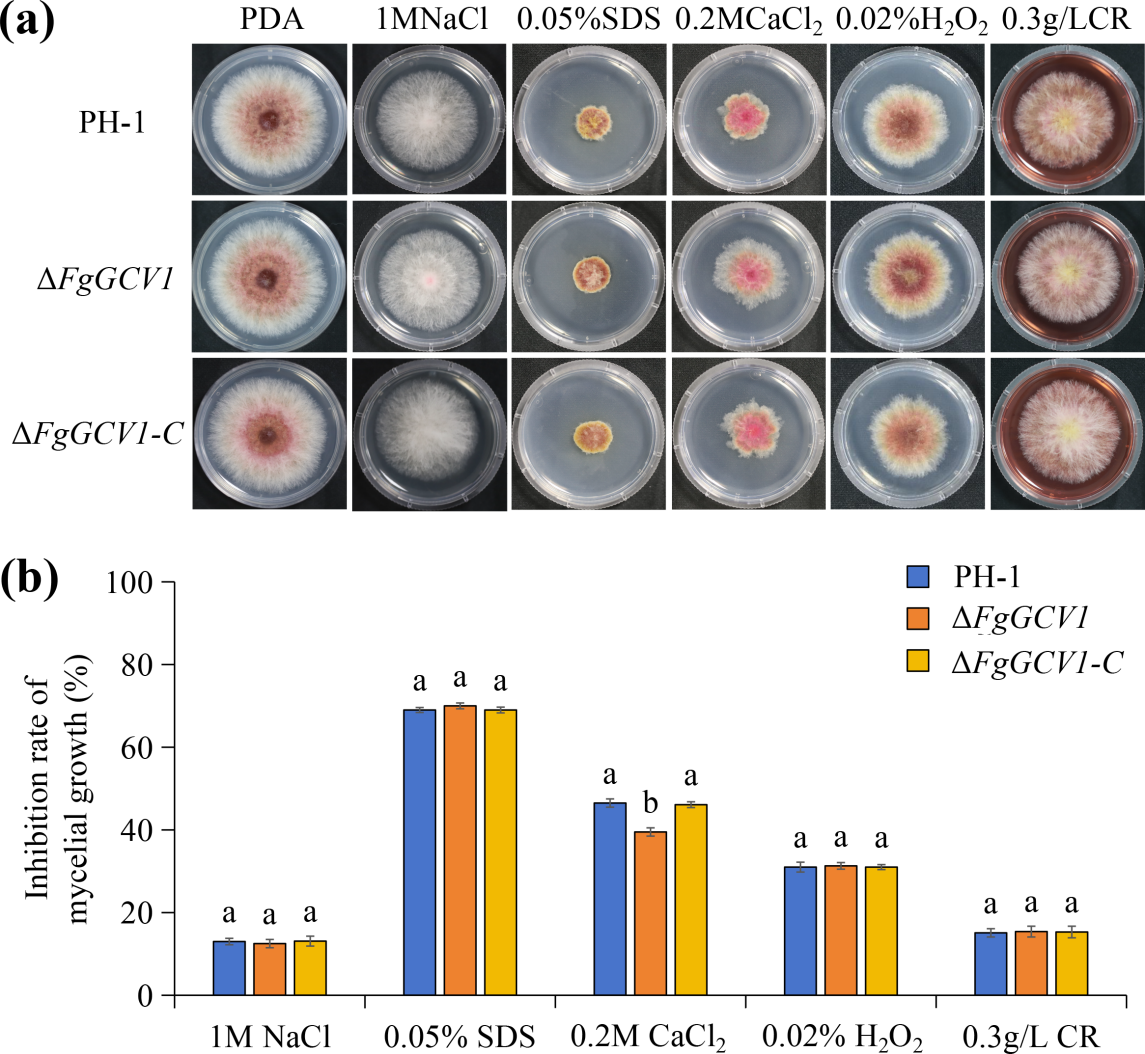
Assays for defects of the Δ*FgGCV1* mutant to various stresses. **(A)** Cultures of the wild-type (PH-1), Δ*FgGCV1* and Δ*FgGCV1-C* strains grown on regular PDA or PDA with 1M NaCl, 0.05% SDS, 0.2M CaCl_2_, 0.02% H_2_O_2_ or 0.3 g/L CR. Photographs were taken after incubation at 25°C for 3 days. **(B)** Percentage inhibition of PH-1, Δ*FgGCV1* and Δ*FgGCV1-C* strains under different stress. Different letters above the bars denote significant differences (*P* < 0.05) by Duncan’s multiple range test.

We next assessed the role of *FgGCV1* in stress tolerance by exposing the wild-type PH-1, Δ*FgGCV1*, and Δ*FgGCV1*-C strains to various stressors. While all strains showed similar sensitivity to hyperosmotic (1 M NaCl), membrane (0.05% SDS), oxidative (0.02% H_2_O_2_) and cell wall (0.3 g/L Congo red) stressors, the Δ*FgGCV1* mutant exhibited significantly increased tolerance to 0.2 M CaCl_2_ (**Figures 5A, B**). This enhanced calcium tolerance was fully restored to wild-type levels in the complemented Δ*FgGCV1*-C strain, indicating a specific role for *FgGCV1* in the fungal response to calcium stress.

### 3.6 FgGCV1-mediated 5,10-CH2-THF metabolism is required for sexual reproduction

To determine whether *FgGCV1* deletion affected glycine metabolism, we quantified the glycine content in wild-type PH-1, Δ*FgGCV1* mutant and Δ*FgGCV1*-C following 48 h of culture at 25°C. The Δ*FgGCV1* mutant exhibited a significant accumulation of glycine (13.63 mg/g), in contrast to WT PH-1 (8.91 mg/g) and Δ*FgGCV1*-C (8.96 mg/g), in which glycine levels were restored to near-WT levels (**Supplementary Figure 4**). This result confirms that *FgGCV1* is essential for normal glycine metabolism in *F. graminearum*.

**Figure 6:**
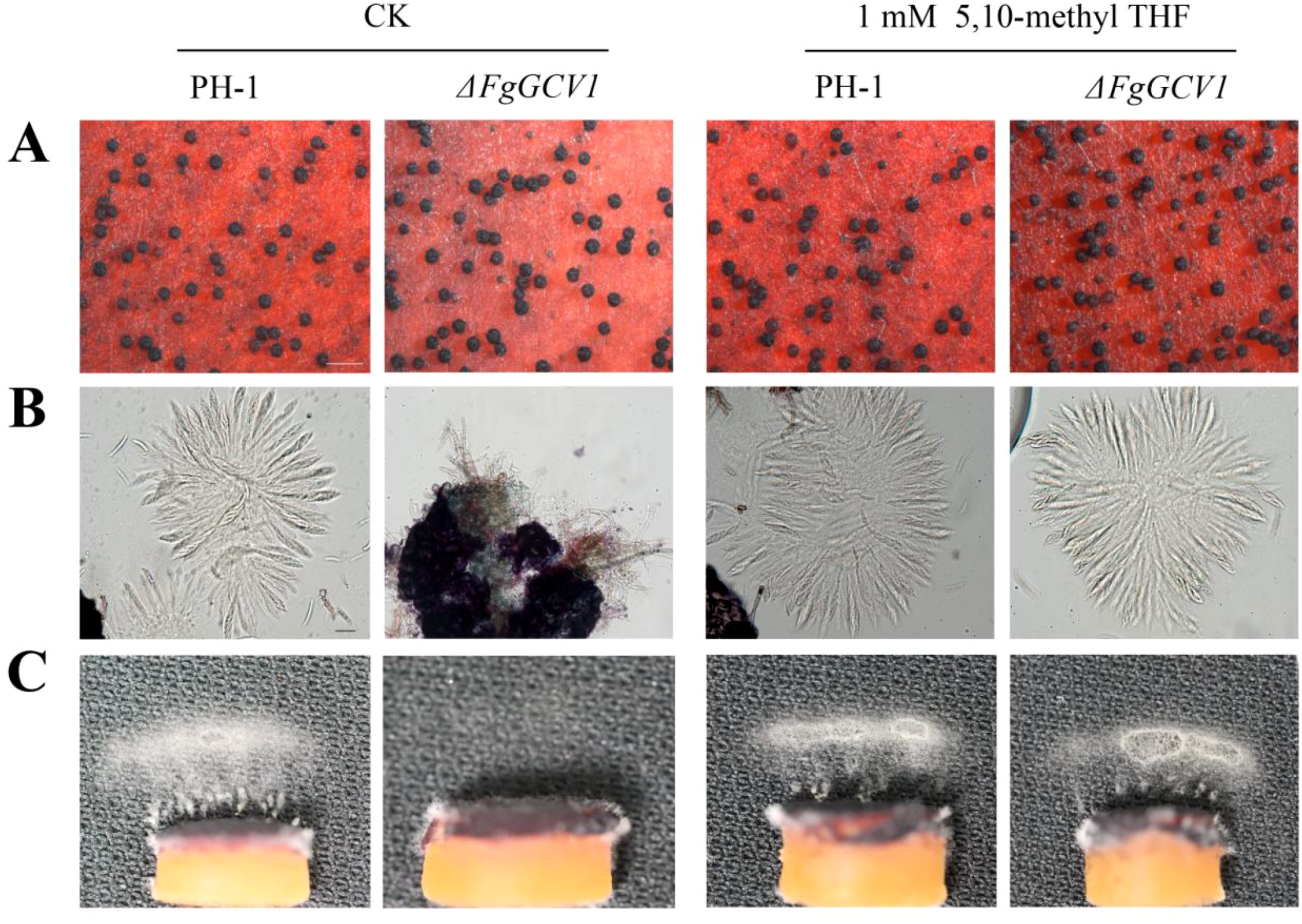
Effect of 5,10-CH_2_-THF on sexual reproduction of Δ*FgGCV1* mutant. Cultures of PH-1, the Δ*FgGCV1* mutant on regular carrot agar (CK) or carrot agar supplemented with 1 mM 5,10-CH_2_-THF were examined for perithecium formation **(A)** and ascus development **(B)** or discharge ascospores **(C)** at 7 d post-fertilization (dpf).

Beyond regulating glycine abundance, the GCS provides one-carbon units derived from glycine to folate one-carbon metabolism (FOCM), which supports downstream processes such as nucleotide biosynthesis and methylation (Fox and Stover, 2008; Leung et al., 2021). We therefore hypothesized that the sexual reproduction defect in the Δ*FgGCV1* mutant results from deficiency in 5,10-CH_2_-THF. To test this, we cultured the Δ*FgGCV1* mutant on carrot agar medium supplemented with 1 mM 5,10-CH_2_-THF (**Figure 6**). The supplementation fully restored the formation of asci and ascospores in the mutant. These results demonstrate that *FgGCV1* regulates sexual development in *F. graminearum* via its role in the GCS to sustain 5,10-CH_2_-THF production.

### 3.7 Transcriptome analysis of the ΔFgGCV1 mutant

**Figure 7:**
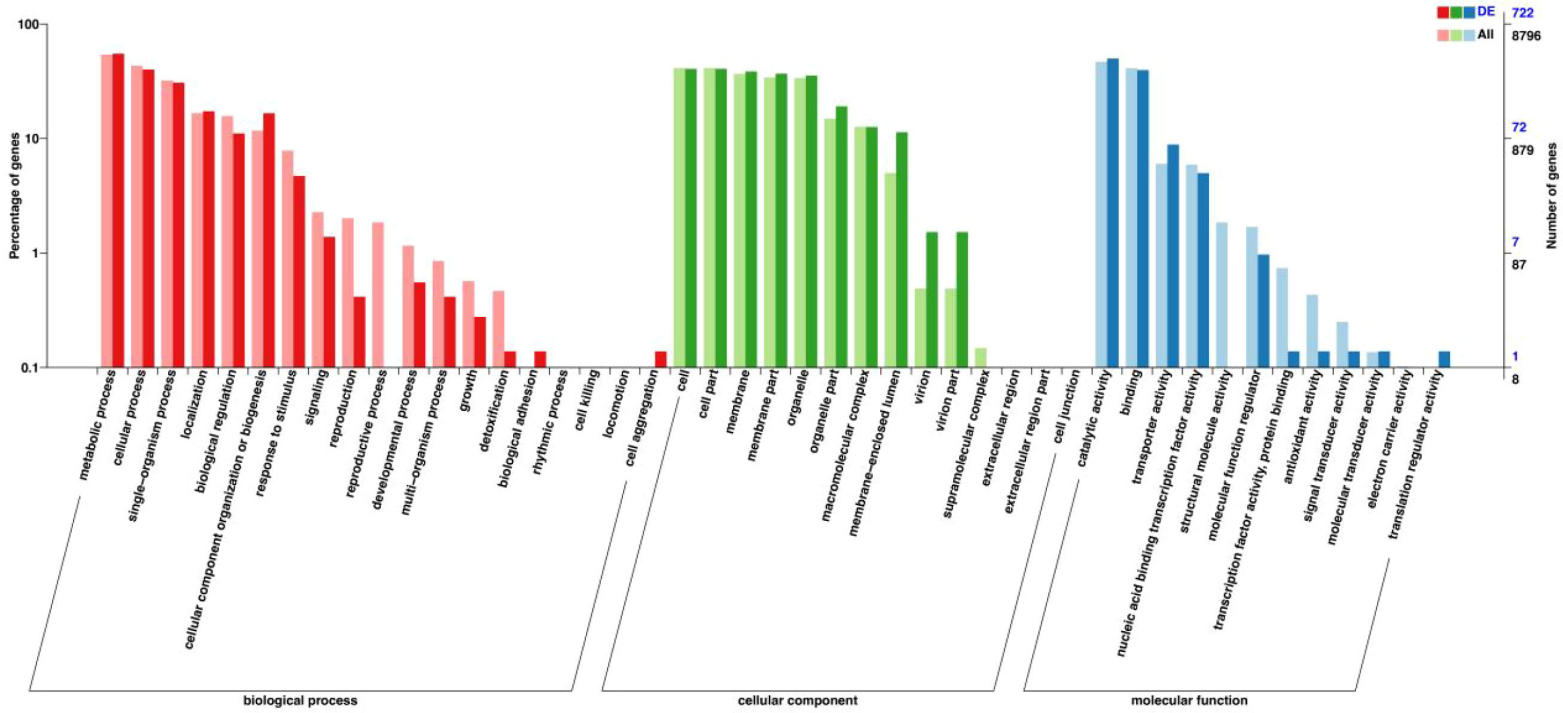
GO enrichment analysis of downregulated DEGs between PH-1 and Δ*FgGCV1* mutant. GO enrichment analysis of DEGs and all genes, classified into three categories: biological process, cellular component, and molecular function. The left y-axis denotes the percentage of genes, while the right y-axis indicates the number of genes. Red and blue bars represent DEGs, whereas pink and green bars correspond to all genes.

To investigate the global transcriptional impact of *FgGCV1* deletion, we performed RNA-seq analysis comparing the Δ*FgGCV1* mutant with wild-type PH-1 strain. A total of 1,482 differentially expressed genes (DEGs) were identified (|log_2_fold-change| > 1, FDR < 0.01), including 976 downregulated and 506 upregulated genes (**Supplementary Figure 5**). These results indicate that *FgGCV1* deletion causes extensive transcriptomic alterations in *F. graminearum*, potentially disrupting multiple biological processes. Gene Ontology (GO) enrichment analysis revealed that the downregulated genes were significantly associated with biological processes such as “cellular component organization or biogenesis”, “response to stimulus”, and “metabolic process”. They were also enriched in cellular components (CCs) including “membrane part”, “organelle”, and “supramolecular complex”, as well as molecular functions (MFs) such as “catalytic activity”, “binding”, and “transporter activity”. Notably, downregulated genes were specifically enriched in reproduction, development, and growth-related pathways (**Figure 7**). In contrast, upregulated genes were mainly involved in biological processes such as “metabolic process”, “single-organism process”, and “cellular component organization or biosynthesis”. Although the enriched CC and MF categories were similar to those of downregulated genes, the enrichment patterns differed, with upregulated genes showing specific associations with “detoxification” and “rhythmic process” (**Supplementary Figure 6**). KEGG pathway analysis further demonstrated that DEGs were significantly enriched in metabolic pathways including “glycine, serine, and threonine metabolism”, “amino acid biosynthesis”, and “carbohydrate metabolism” (**Supplementary Figure 7**). Importantly, several genes involved in glycine, serine, and threonine metabolism showed significant expression changes. A set of these genes (FGSG_02279, FGSG_02271, FGSG_07266, FGSG_06544, FGSG_10743, FGSG_11228, FGSG_00296, FGSG_10119, FGSG_03278, FGSG_10677) were selected for RT-qPCR validation (**Supplementary Figure 8**). Their expression levels were significantly downregulated in Δ*FgGCV1* compared to WT PH-1, which is consistent with the RNA-seq results. This not only confirms the reliability of our transcriptome data but also directly implicates *FgGCV1* in the regulation of glycine, serine, and threonine metabolism in *F. graminearum.* Collectively, these transcriptomic findings suggest that *FgGCV1* deletion predominantly affects genes related to reproduction and development in *F. graminearum*, and that *FgGCV1* regulates physiological processes largely through modulating glycine, serine, and threonine metabolism.

## 4 Discussion

As the core enzyme of the glycine cleavage system (GCS), the T protein (GCVT) plays an irreplaceable role in one-carbon metabolism, energy conversion and nitrogen balance (Ren et al., 2022). In this study, we identified two GCVT homologs, FgGCV1 and FgGCV2, in the genome of *F. graminearum* strain PH-1. Bioinformatic analyses showed that FgGCV1 is highly conserved between *F. graminearum* and *S. cerevisiae* (**Supplementary Figure 9**). The FgGCV2 contains a Glycine/D-amino acid oxidase domain, an FAO_M domain, and a GCVT protein domain (**Figure 2A**), and its orthologs are well conserved in *Fusarium*, including *Fusarium verticillioides*, *Fusarium oxysporum*, *Fusarium solani* and *Fusarium pseudograminearum* (**Figure 1**). The absence of a distinct *FgGCV2* ortholog in *S. cerevisiae* suggests that this gene may have been lost in certain *Saccharomycetales* species during evolution. Consistent with the established mitochondrial role of GCS in carbon-nitrogen metabolism in eukaryotes (Kikuchi & Hiraga, 1982; Okamura-Ikeda et al., 1991; Vauclare et al., 1998; Lee et al., 2004), both FgGCV1 and FgGCV2 localized to mitochondria (**Figure 3**).

The GCS serves as the principal pathway for glycine degradation, and its dysfunction typically leads to impaired glycine catabolism and intracellular accumulation (Ichinohe et al., 2004; Leung et al., 2020). In humans, mutations in the GCS component *AMT* cause glycine accumulation and disrupt folate-mediated one-carbon metabolism (FOCM), leading to neurometabolic disorders and developmental brain defects such as neural tube defects (NTDs) and ventriculomegaly (Hoover-Fong et al., 2004; Swanson et al., 2015; Leung et al., 2021). Similarly, *Gldc-*deficient mice show loss of GCS activity and elevated glycine and glycine derivatives in plasma and tissues (Pai et al., 2015; Leung et al., 2020). In *S. griseus*, deletion of the *gcvT* gene also results in complete loss of the GCV activity and intracellular glycine accumulation (Tezuka et al., 2014). Consistent with these findings, we observed a significant increase in total glycine content in the Δ*FgGCV1* mutant compared to the wild-type PH-1 (**Supplementary Figure 4**), confirming the observed role of *FgGCV1* in glycine metabolism in *F. graminearum*.

Notably, during sexual reproduction, the expression of *FgGCV1* was upregulated, whereas *FgGCV2* was downregulated (**Figure 2C**), implying a specific role for *FgGCV1* in this process. Indeed, while deletion of *FgGCV2* has no discernible phenotypic effect, the Δ*FgGCV1* mutant exhibited severe defects in asci development and ascospore formation (**Figure 4**). A key mechanistic insight came from the rescue of this defect by supplementation with 5,10-CH_2_-THF, a key one-carbon unit carrier in the GCS, which fully restored the sexual reproduction in the Δ*FgGCV1* mutant (**Figure 6**). This confirms that *FgGCV1* regulates sexual reproduction through glycine and one-carbon metabolism. Sexual reproduction in fungi is an energy-demanding process that relies heavily on amino acids as nutrients (Son et al., 2011). Previous studies have shown that genes involved in amino acid metabolism, such as *GzmetE* involved in methionine biosynthesis (Wang et al., 2021) and transcription factors regulating nitrogen metabolism (Hou et al., 2015), are essential for sexual development in *F. graminearum*. Our results further highlight the critical role of the glycine metabolism as a carbon and one-carbon source in powering sexual reproduction in *F. graminearum*.

Calcium signaling is a major regulator of morphogenetic and physiological processes in filamentous fungi (Kurian et al., 2022). In *F. graminearum*, turgor pressure in asci-driven by ion fluxes including K^+^, Na^+^, Cl^-^ and Ca^2+^ is essential for ascospore discharge (Trail et al., 2002; Trail, 2007; Min et al., 2010). Interestingly, the Δ*FgGCV1* mutant exhibited significantly reduced growth inhibition under 0.2 M CaCl_2_ stress compared to wild-type PH-1 and Δ*FgGCV1*-C strains (**Figure 5**), suggesting a role for *FgGCV1* in calcium stress response that may be linked to its reproductive function.

Transcriptome analysis revealed extensive gene expression changes in the Δ*FgGCV1* mutant, with 1,482 differentially expressed genes (**Supplementary Figure 5**). Among these, key genes in glycine, serine, and threonine metabolism—such as *FGSG_07266* (encoding 5-aminolevulinate synthase) and *FGSG_10119* (encoding threonine dehydratase)—were markedly downregulated (**Supplementary Figure 8**). These enzymes are critical for serine biosynthesis and glycine utilization, supporting a central role for *FgGCV1* in modulating this metabolic axis. KEGG analysis further indicated that *FgGCV1* deletion affects broader metabolic pathways, including amino acid biosynthesis and carbohydrate metabolism (**Supplementary Figure 7**), consistent with the role of GCVT as a metabolic hub in carbon-nitrogen interplay (Merrick and Edwards, 1995; Douce et al., 2001).

In conclusion, we have characterized the biological functions of two GCVT homologs (*FgGCV1* and *FgGCV2*) in *F. graminearum* and demonstrated that *FgGCV1* but not *FgGCV2* is essential for sexual reproduction. The developmental defect in Δ*FgGCV1* stems from disrupted one-carbon metabolism, as evidenced by the phenotypic rescue with 5,10-CH_2_-THF supplementation. The Δ*FgGCV1* mutant also exhibited altered calcium stress sensitivity and significant transcriptional reprogramming of metabolic genes. To our knowledge, this is the first study to functionally link a GCVT homolog in ascomycete fungi to the regulation of folate-mediated one-carbon metabolism and sexual development. Future work should focus on identifying additional components of glycine cleavage system and elucidating the regulatory networks that integrate glycine metabolism with development and stress adaptation in filamentous fungi.

## Data Availability

The datasets presented in this study can be found in online repositories. The names of the repository/repositories and accession number(s) can be found in the article/**Supplementary Material**.
