# Supplementary figures and images for: FgGCV1, a glycine cleavage system T protein, regulates glycine metabolism and sexual reproduction in Fusarium graminearum
Source: Front Plant Sci. 2026 Mar 18;17:1771151. doi: 10.3389/fpls.2026.1771151 (PMC13038437; doi:10.3389/fpls.2026.1771151)

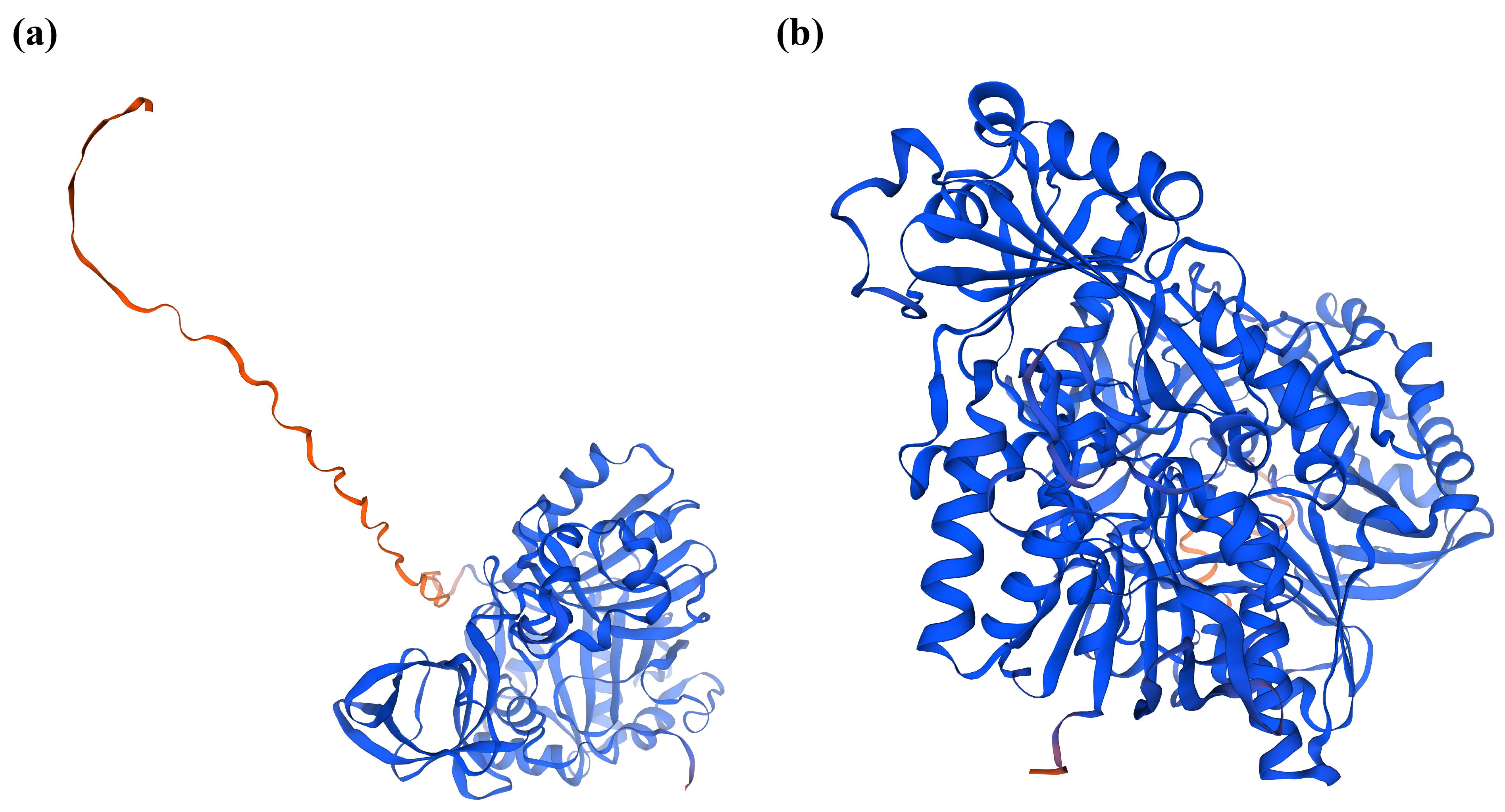

Supplement: Supplementary file 1 [file Image1.jpeg]

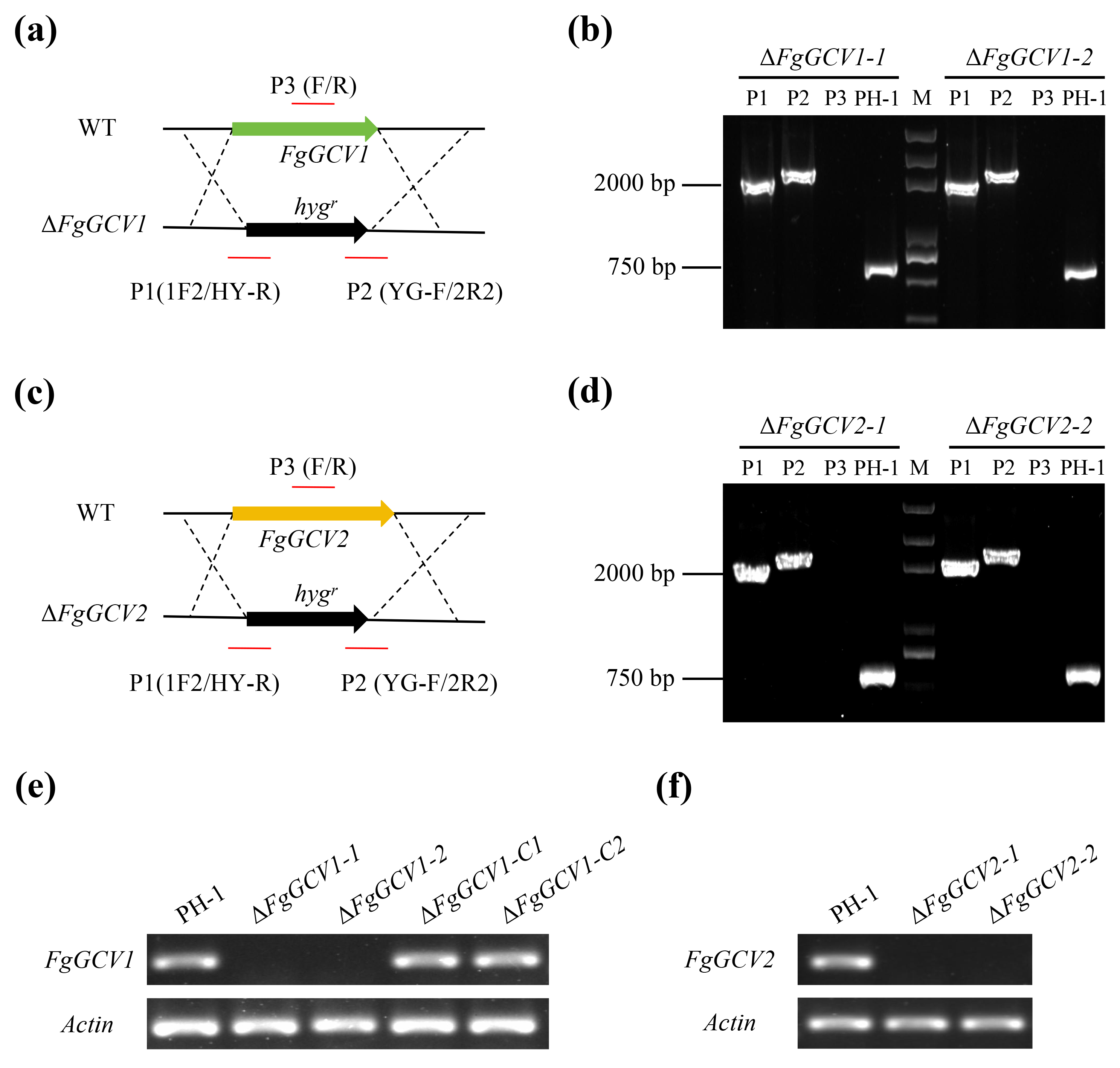

Supplement: Supplementary file 2 [file Image2.jpeg]

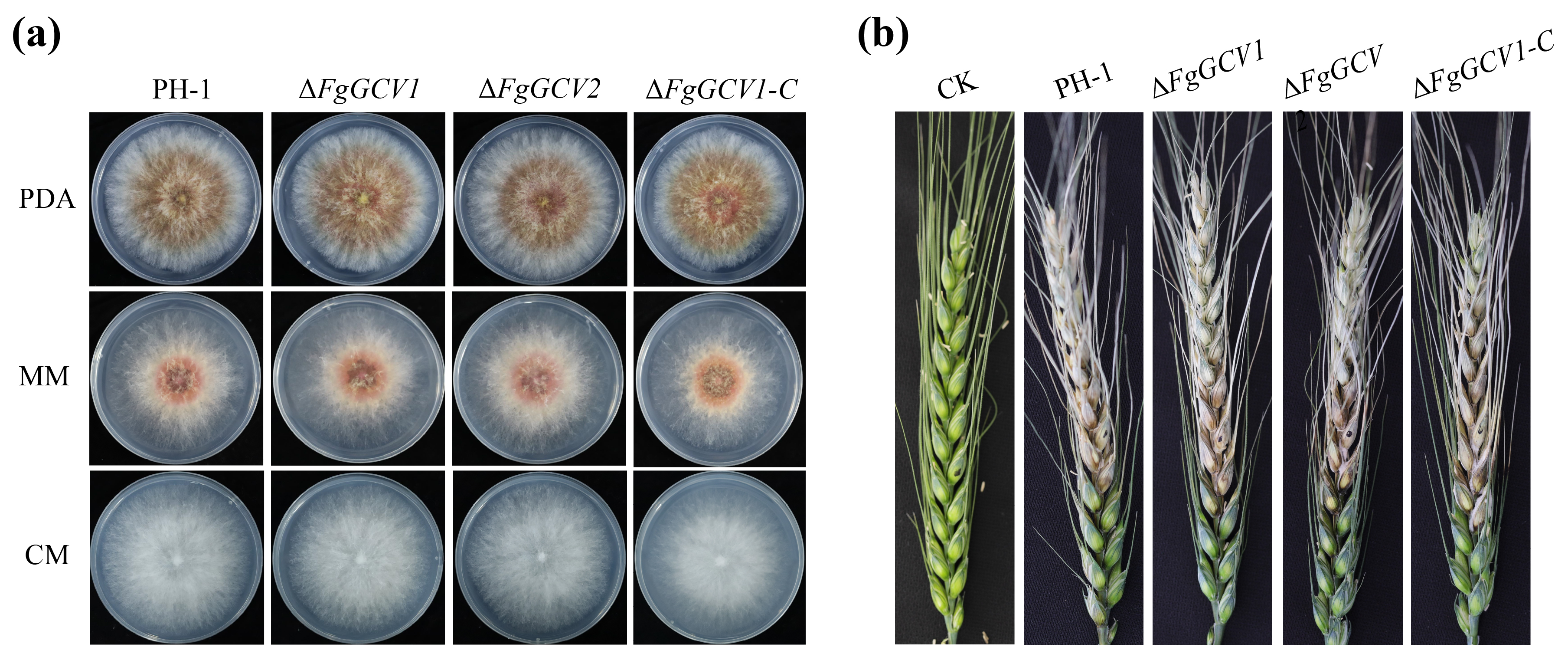

Supplement: Supplementary file 3 [file Image3.jpeg]

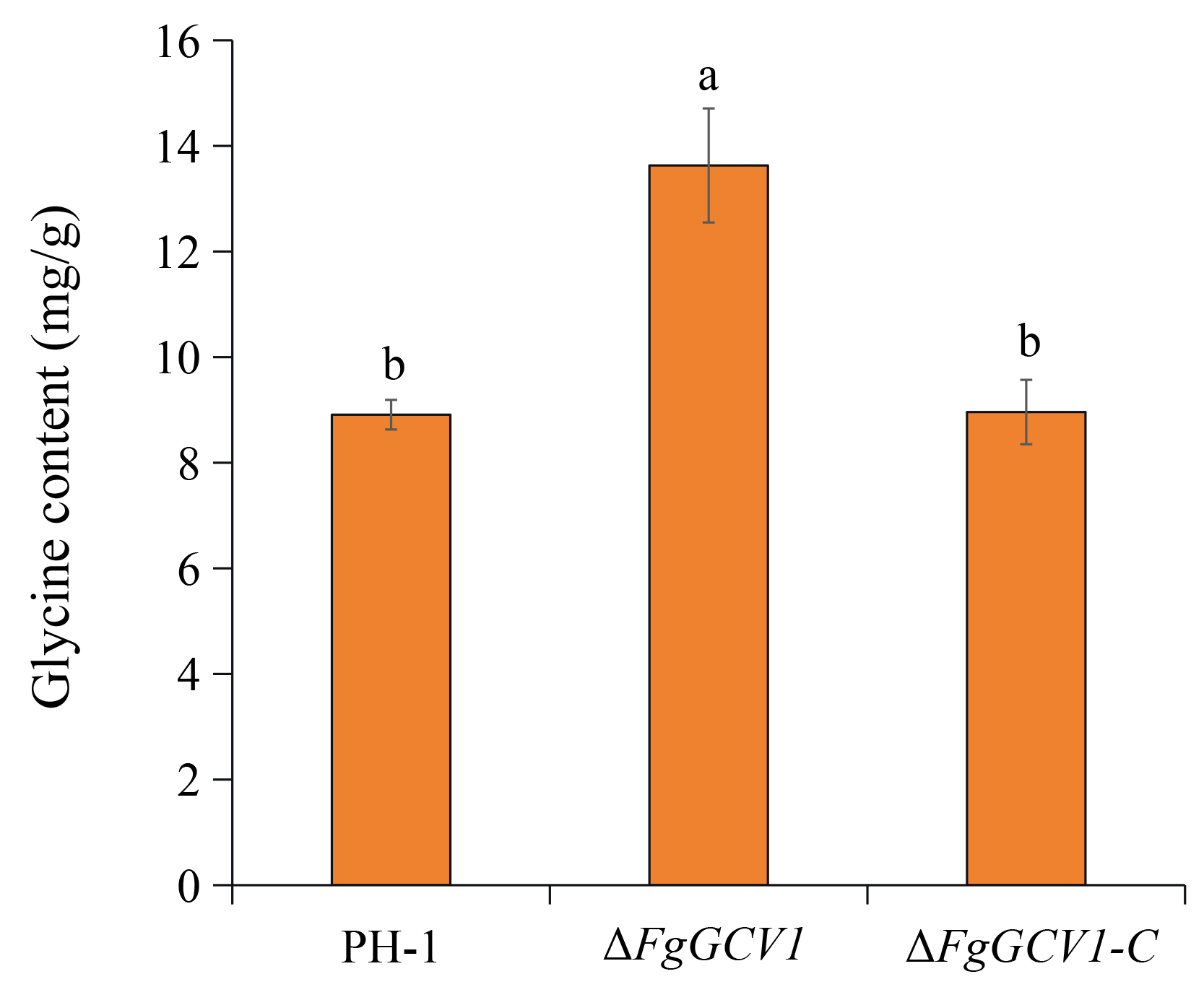

Supplement: Supplementary file 4 [file Image4.jpeg]

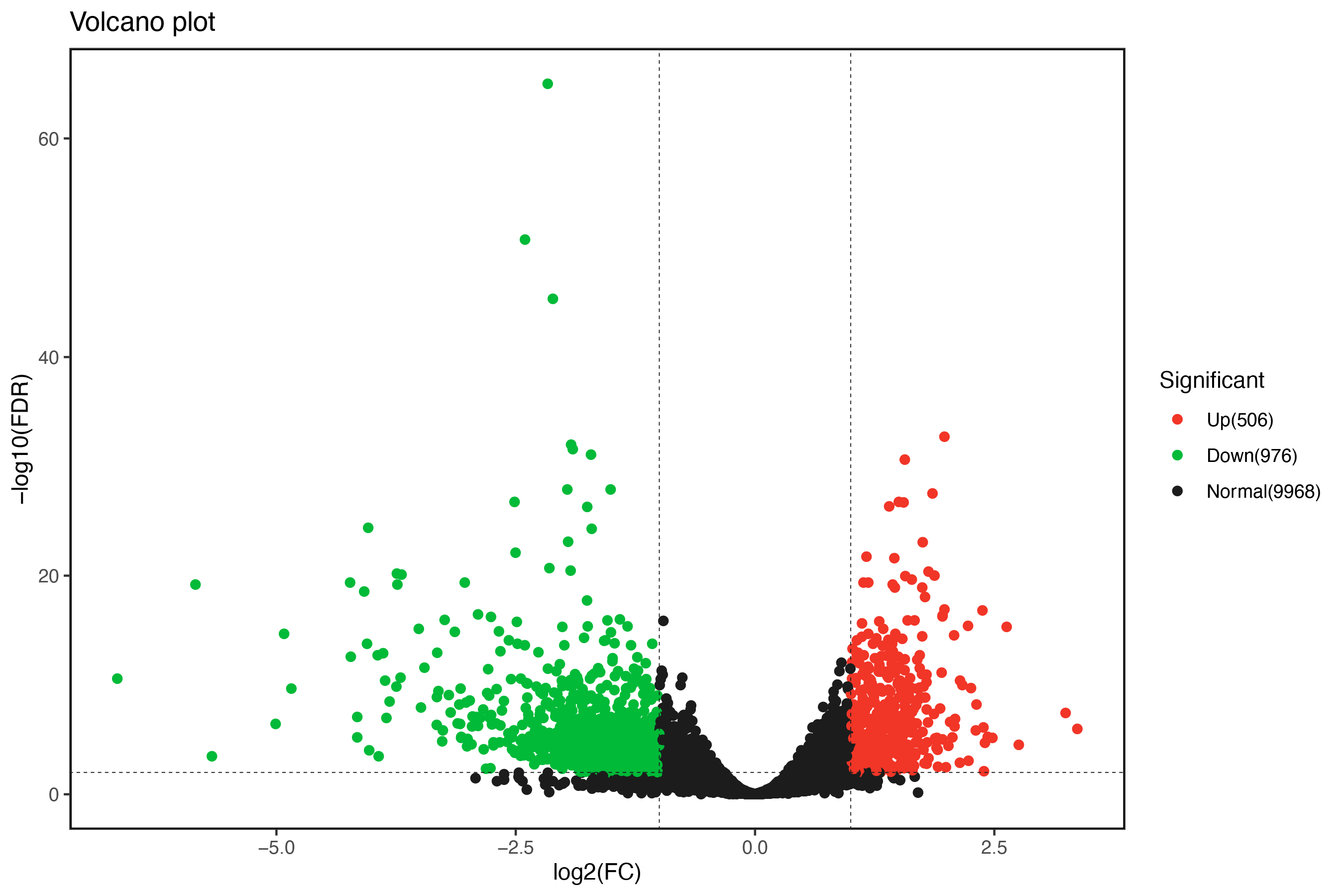

Supplement: Supplementary file 5 [file Image5.jpeg]

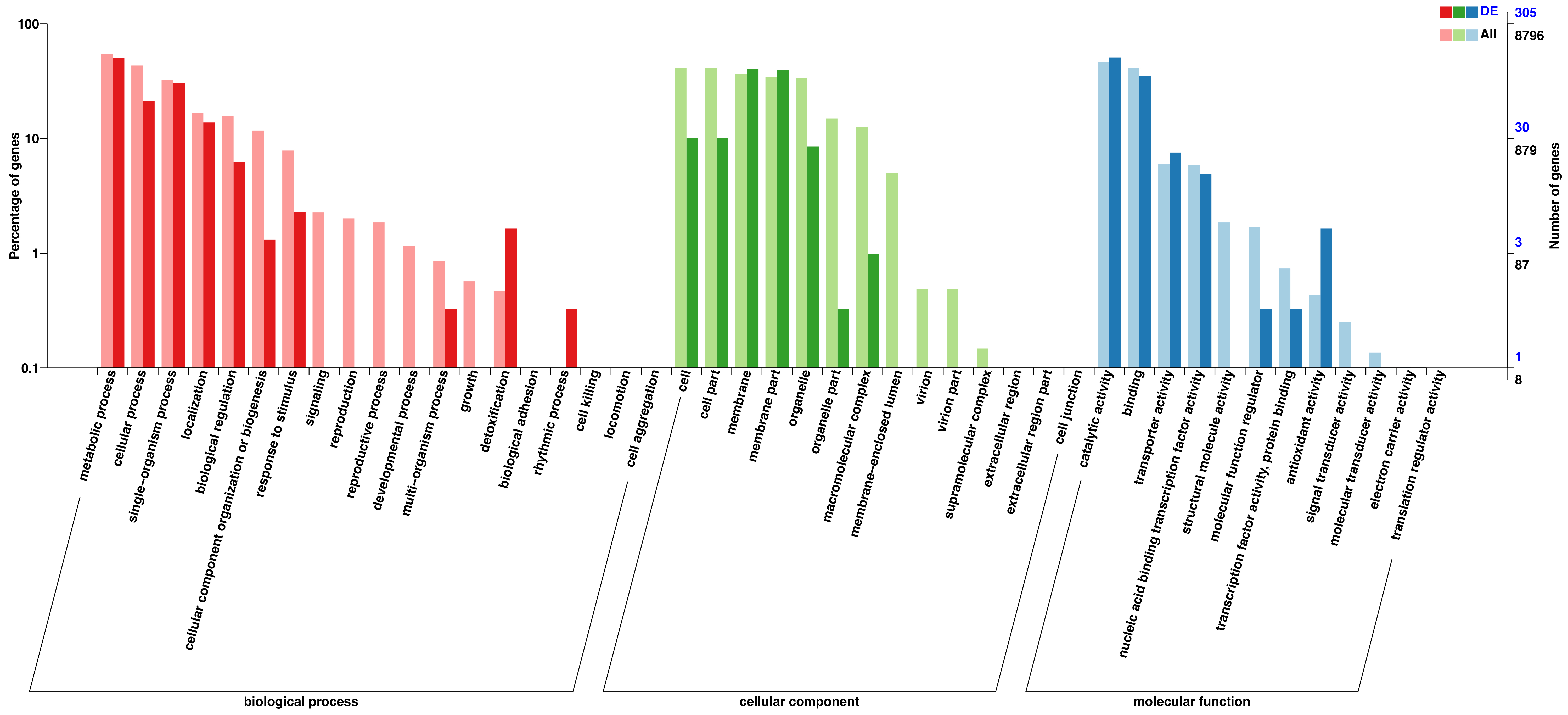

Supplement: Supplementary file 6 [file Image6.jpeg]

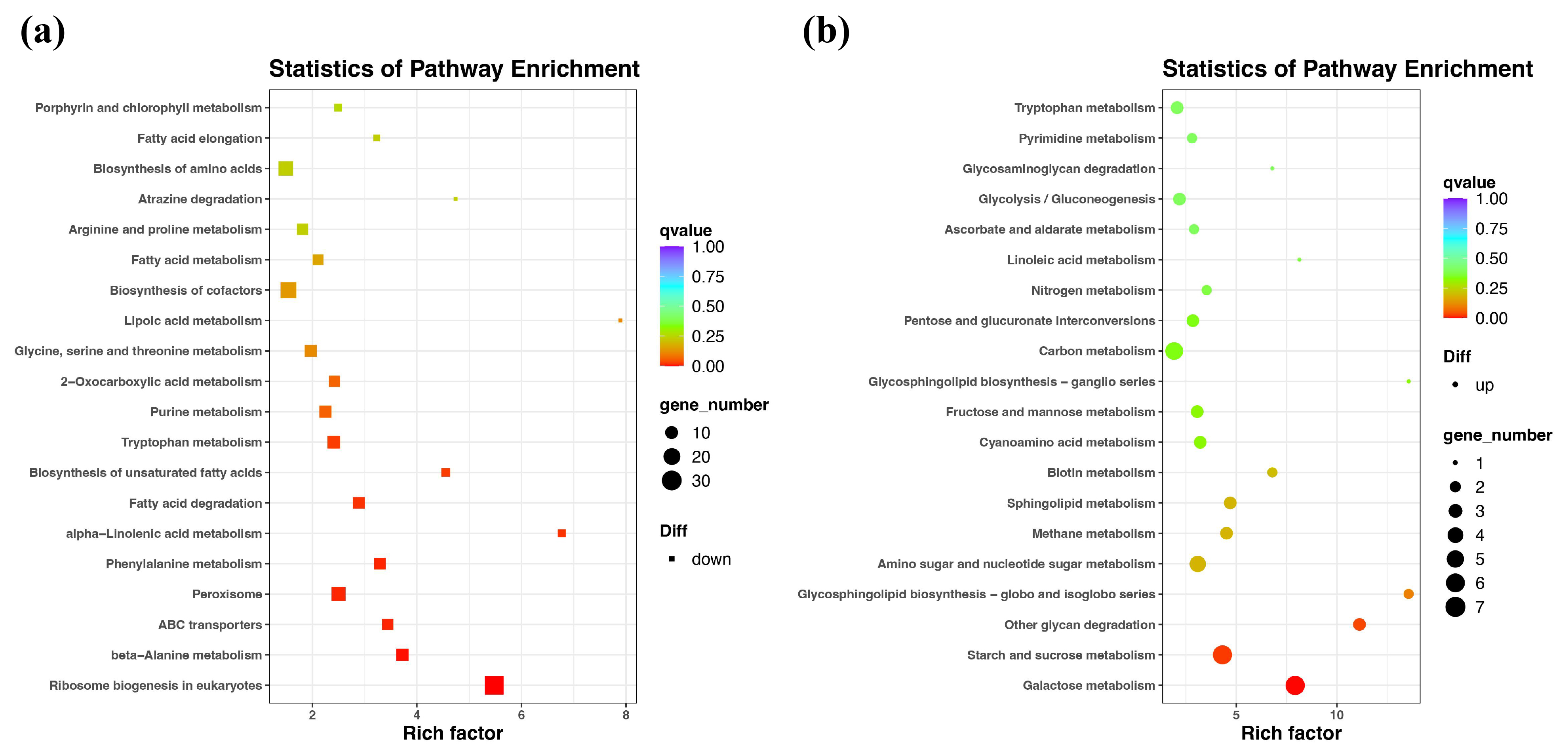

Supplement: Supplementary file 7 [file Image7.jpeg]

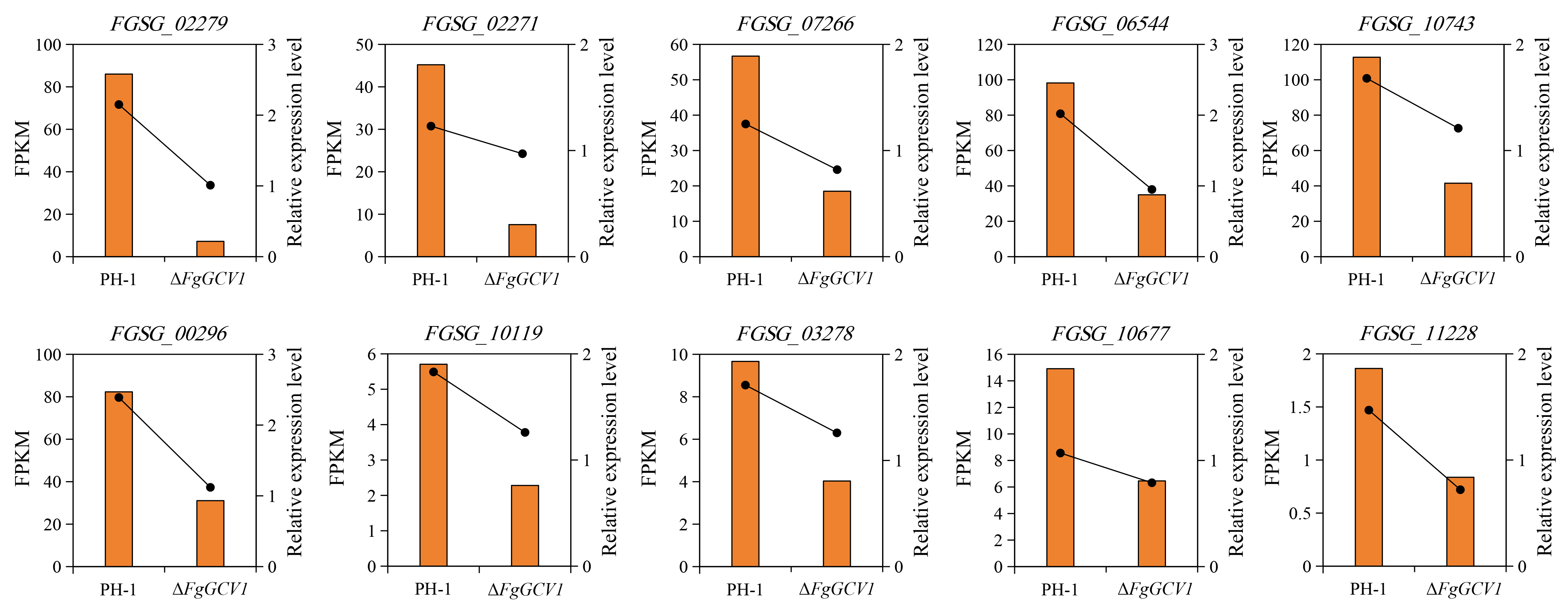

Supplement: Supplementary file 8 [file Image8.jpeg]

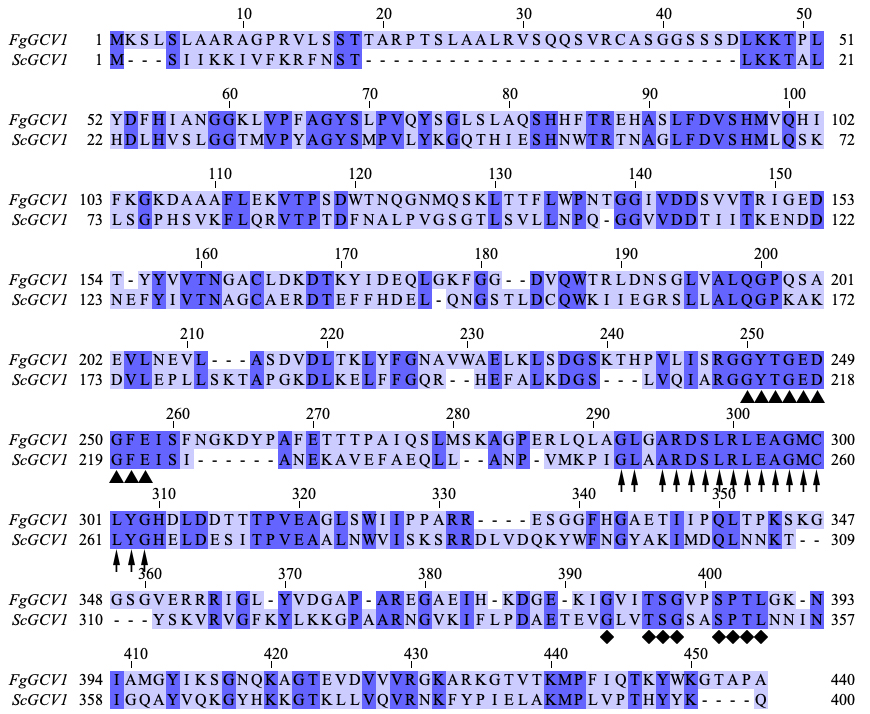

Supplement: Supplementary file 9 [file Image9.jpeg]
