# Supplementary material for: FgGCV1, a glycine cleavage system T protein, regulates glycine metabolism and sexual reproduction in Fusarium graminearum
Source: Front Plant Sci. 2026 Mar 18;17:1771151. doi: 10.3389/fpls.2026.1771151 (PMC13038437; doi:10.3389/fpls.2026.1771151)
Supplement: Supplementary file 13 [file Table4.docx]

Supplementary Material

# Supplementary Figures and Tables

## Supplementary Figures

**Supplementary Figure 1.** Tertiary structures of FgGCV1 (a) and FgGCV2 (b) in *F. graminearum*. Tertiary structures were predicted using AlphaFold v2 and visualized using UCSF Chimera.

**Supplementary Figure 2.** Generation and verification of Δ*FgGCV1* and Δ*FgGCV2* mutants. (a) Schematic diagram of *FgGCV1* replacement strategy. hyg^r^, hygromycin B resistance gene cassette; P1 (primer pair 1F1/1R): the fragment P1, which was used to identify the upstream homologous recombination of the deletion mutant; P2 (primer pair 2F/2R1): the fragment P2, which was used to identify the downstream homologous recombination of the deletion mutant; P3 (primer pair F/R): the fragment P3, which was used to identify deletion mutant. The red lines indicate the region of PCR amplification with primers. (b) Verification of *FgGCV1* deletion mutants using PCR. P1, P2, and P3 indicate fragments P1, P2, and P3, respectively. M, DL5000 Marker. (c) Schematic diagram of *FgGCV2* replacement strategy. (d) Verification of *FgGCV2* deletion mutants. (e-f) The Δ*FgGCV1*, Δ*FgGCV2* and Δ*FgGCV1*-C strains were confirmed by RT-qPCR.

**Supplementary Figure 3.** The assays of the mycelial growth and virulence of PH-1, Δ*FgGCV1*, Δ*FgGCV2* and Δ*FgGCV1-C* strains. (a) Colony morphology of the different strains on potato dextrose agar (PDA), minimal medium (MM), and complete medium (CM) plates, respectively. Strains were cultured at 25°C for 3-4 days and photographed. (b) Wheat spikelets were inoculated with the WT PH-1, Δ*FgGCV1*, Δ*FgGCV2* and Δ*FgGCV1-C* strains, and photographed at 14 dpi.

**Supplementary Figure 4.** The Δ*FgGCV1* mutant is defective in glycine content. Assessment of the total glycine content in wild-type PH-1, Δ*FgGCV1* and Δ*FgGCV1-C* strains. The different lowercase letters in the bars represent significant differences according to the Duncan's multiple range test at *P* < 0.05.

**Supplementary Figure 5.** Volcano plot of significantly different genes in the Δ*FgGCV1* mutant. The numbers of upregulated (red dots) and downregulated (green dots) genes (log_2_FC > 1, FDR < 0.01) with standard deviation were calculated with data from three biological replicates.

**Supplementary Figure 6.** GO terms enrichment analysis for significantly upregulated genes.

**Supplementary Figure 7.** KEGG enrichment analysis of the upregulated and downregulated genes in the Δ*FgGCV1* strain. (a) Top KEGG pathway terms for significantly downregulated genes. (b) Top KEGG pathway terms for significantly upregulated genes.

**Supplementary Figure 8.** Expression profiles of selected genes in the wild-type PH-1 and Δ*FgGCV1* strains. Each subpanel depicts the expression level of an individual gene (FGSG_02279, FGSG_02271, FGSG_07266, FGSG_06544, FGSG_10743, FGSG_00296, FGSG_10119, FGSG_03278, FGSG_10677 and FGSG_11228) measured by RNA-seq (FPKM, left y-axis, orange bars) and RT-qPCR (right y-axis, black dots connected by lines).

**Supplementary Figure 9.** Amino acid sequence alignment between *S. cerevisiae* and *F. graminearum*. Bold triangles, arrows and rhombuses indicate the TGYTGEXGXE motif, PXGLGARDXXRhEAXXXLYG motif, and GXh(T/S)(S/T)GXXSPTL motif, respectively.

## Supplementary Tables

**Supplementary Table 1.** Protein properties of two GCVT genes in *F. graminearum*.

**Supplementary Table 2.** Growth rate, conidiation, and virulence of different *F. graminearum* strains.

**Supplementary Table 3.** Primers used in this study.
